# The effect of PBL teaching method in the teaching of congenital malformation

**DOI:** 10.3389/fmed.2025.1508415

**Published:** 2025-05-14

**Authors:** Hua Li, Zejun Cai, Yue Liu, Yong Chen, Qiaomei Lin, Hui Liu

**Affiliations:** ^1^Department of Histology and Embryology, School of Basic Medical Sciences, Fujian Medical University, Fuzhou, Fujian, China; ^2^Key Laboratory of Stem Cell Engineering and Regenerative Medicine of Fujian Province University, School of Basic Medical Sciences, Fujian Medical University, Fuzhou, Fujian, China

**Keywords:** PBL teaching method, traditional teaching methods, congenital malformation, questionnaire survey, evaluation

## Abstract

**Objective:**

To explore the effect of PBL (Problem-Based Learning) teaching method in the teaching of congenital malformation.

**Methods:**

The application of PBL teaching method in the teaching process of congenital malformations among clinical medicine majors in grades 2021 and 2022. And the effect of PBL teaching method in teaching congenital malformations was analyzed through pre-class intra-group evaluation, in-class inter-group evaluation, and post-class questionnaire survey.

**Results:**

There was a significant difference in inter-group evaluation scores during class, and a post-class questionnaire survey showed that students have made a qualitative leap in their understanding of congenital malformations after PBL teaching.

**Conclusion:**

The PBL teaching method enhances students’ teamwork ability, stimulates their innovative thinking, and enhances their interest in learning.

## Introduction

Congenital malformations refer to abnormalities in morphology or structure that occur during embryonic development (1, 2). Most congenital malformations are characterized by high mortality rates and poor prognosis, serving as one of the key reasons for spontaneous abortions or stillbirths in pregnant women, and also the primary cause of disabilities in children (2–5). Since China implemented the three-child policy in 2021 (6), there has been a significant increase in the number of older pregnant women, which undoubtedly has led to a relative rise in the number of malformed fetuses in our country (6). The government attaches great importance to the prevention and treatment of congenital malformations. In 2005, the Chinese government declared that September 12 would annually be “National Birth Defects Prevention Day” (NBDPD), actively promoting awareness of congenital malformation prevention among the general public while strengthening talent cultivation. By imparting knowledge and skills related to congenital malformations to medical professionals, a foundation is laid for enhancing prevention and treatment capabilities in the future. Nevertheless, traditional teaching methods for congenital malformations tend to be teacher-centered, with students passively receiving information, leading to a lack of active thinking and exploration abilities.

Problem-Based Learning (PBL), also known as question-based learning, involves medical students engaging in discussions centered around a specific topic or case study under the guidance of teachers, typically in the form of group discussions (7–14). In the PBL teaching model, teachers shift from being sole providers of knowledge to acting as mentors and supporters, guiding the learning process and providing necessary support and feedback. Students, on the other hand, transition from passive recipients of information to active learners, solving problems through self-inquiry and collaborative learning. This forms a collaborative relationship between teachers and students, enhancing classroom learning.

This article explores the effectiveness of PBL in teaching congenital malformations by implementing this methodology in congenital malformation courses for students in the 2021 and 2022 clinical medicine programs at Fujian Medical University.

## Methods

### Teaching implementation

The instruction for medical students majoring in clinical medicine was structured into two phases. The study population consisted from the 2021 and 2022 cohorts (age range 19-21 years; 55% female, 45% male), all of whom had completed fundamental courses in anatomy, cell biology, physiology, statistics, immunology, and pathology prior to participation. The first phase involves classroom lectures in the second semester of the freshman year, while the second phase utilizes PBL methodology for discussion-based teaching in the first semester of the sophomore year (4 h/2 sessions). During the first PBL session, the primary tasks are for teachers to present relevant case studies and for students to engage in group discussions. The case studies center around three scenarios: antenatal checkups for pregnant women, auxiliary examinations, and childbirth. Through each scenario, accompanied by learning objectives and questions, students engaged in group discussions to jointly explore issues such as the causes of fetal malformation, diagnostic methods, and potential management of doctor-patient relationships. During this process, the teacher actively guided the discussions, providing necessary support and feedback, ultimately setting the themes for the groups’ presentations in the second session.

**Figure 1:**
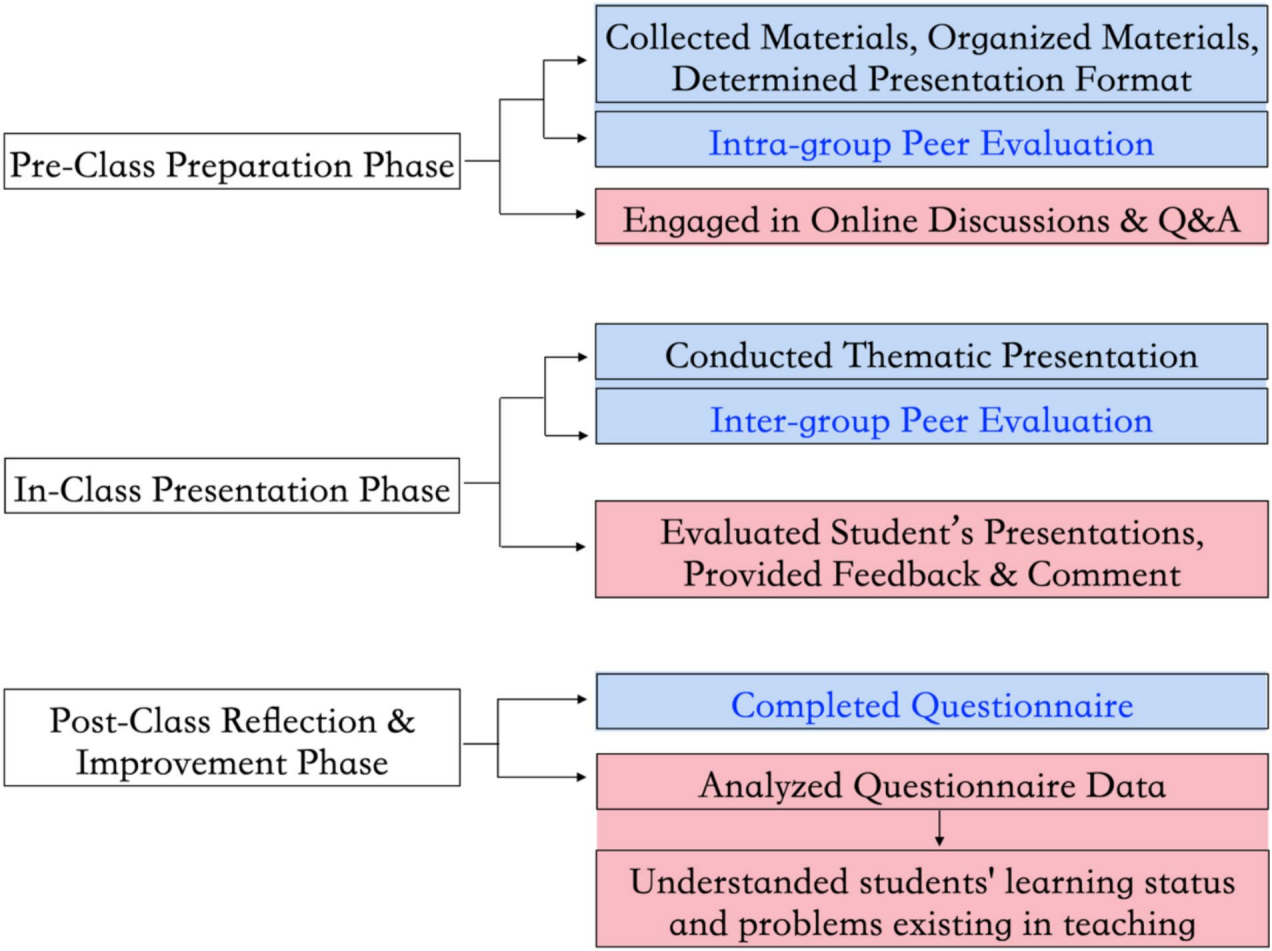
The flowchart demonstrates the second session of the PBL teaching process. In the chart, operations within the blue boxes belong to students, while those within the red boxes belong to teachers. The assessment process in which students participate is indicated by the blue font.

After the first session, each group’s chairperson led the secretaries and reporters in preparing their presentation content based on the assigned themes and internal roles. This involved researching materials, resolving questions, determining the presentation format, and conducting peer evaluations within the group. Meanwhile, the teacher provided assistance through online platforms like Superstar or QQ, answering questions and ensuring students completed their preparation on time (15–17). During the second session, each group presented their work, either through role-playing scenarios, video presentations, PowerPoint demonstrations, or proposals for practical activities promoting eugenics and prenatal care. Following the presentations, students conducted peer evaluations among groups. Finally, the teacher provided comments and a summary. After class, students completed a questionnaire related to the PBL teaching methodology designed by the teacher, and the teacher analyzed the questionnaire results statistically to understand students’ learning progress. The Figure 1 illustrates the second session flowchart of the PBL teaching process.

### Evaluation method

Employing a diversified evaluation approach that encompasses pre-class peer evaluation within groups, in-class peer evaluation between groups, and post-class questionnaires, allows students to fully participate in the evaluation process (18–20). To ensure objectivity, all peer evaluations were conducted using a blinded review system (anonymous assessment), and faculty members independently scored each group using the same standardized criteria. The scores from pre-class and in-class peer evaluations are included in the students’ overall course grades.

This scoring mechanism is designed with clear and precise evaluation criteria to ensure objectivity and feasibility. The assessment is divided into two main components: pre-class peer evaluation within groups and in-class peer evaluation between groups, each with specific scoring guidelines. The detailed criteria and point ranges are outlined in the table below (Table 1).

**Table 1:** Evaluation criteria table.

Evaluation phase	Evaluation type	Scoring criteria	Point range	Description
Pre-class Evaluation	Intra-group Peer Review	*Contribution to Task Completion*: The extent to which the member actively participated in completing assigned tasks.	0–25 points	Prevents free-riding, ensures accountability, and fosters a collaborative and productive team environment.
		*Quality of Work*: The accuracy, depth, and creativity of the member’s contributions.	0–25 points	Evaluates whether the member’s work meets expectations and demonstrates innovation.
		*Collaboration and Communication*: The member’s ability to communicate effectively and collaborate with the team.	0–25 points	Assesses the member’s teamwork skills, including communication and cooperation.
		*Timeliness*: Whether the member met deadlines and adhered to the group’s schedule.	0–25 points	Ensures tasks are completed on time, avoiding delays that could impact the team’s progress.
In-class Evaluation	Inter-group Peer Review	*Content Quality*: The depth, relevance, and accuracy of the presented material.	0–30 points	Evaluates whether the content is substantial, relevant, and accurate, effectively conveying ideas.
		*Presentation Format*: The organization, clarity, and visual appeal of the presentation.	0–20 points	Assesses the structure, clarity, and visual attractiveness of the presentation.
		*Delivery and Engagement*: The presenter’s ability to engage the audience, articulate ideas clearly, and respond to questions effectively.	0–20 points	Evaluates the presenter’s delivery skills and interaction with the audience.
		*Overall Effectiveness*: The overall impact and persuasiveness of the presentation.	0–30 points	Provides a comprehensive assessment of the presentation’s overall performance.
Additional Requirement	Written Feedback	Students are required to provide written comments alongside their scores, highlighting strengths and areas for improvement.	-	Encourages critical thinking, deepens understanding of the content, and provides constructive feedback to peers.

In summary, this diversified evaluation approach not only ensures fairness and objectivity in assessing student performance but also fosters an environment conducive to active learning, teamwork, and continuous improvement.

### Data statistics and analysis methods

This study employed multiple assessment methods to collect data, including intra-group peer evaluation, inter-group peer evaluation, and questionnaires survey. Intra-group peer evaluation focused on the performance of team members during collaborative learning activities, while inter-group peer evaluation assessed the collaboration and competition dynamics between different groups. Questionnaires survey were used to gather students’ feedback on teaching methods, learning outcomes, and course satisfaction. To ensure the comprehensiveness and representativeness of the data, all assessment methods were implemented across multiple classes and conducted repeatedly to capture diverse teaching scenarios and student perspectives.

Differences in the inter-group peer evaluation score were analyzed using Student’s t-test in GraphPad Prism7; results are expressed as mean ± standard deviation (SD), with a significance level established at *p* < 0.05. Both Cronbach’s alpha (for reliability) and chi-square tests (for comparative analysis) were conducted using SPSS.

## Results

### Intra-group peer evaluation

The results of intra-group peer evaluation showed no significant differences, indicating that group members recognized each other’s earnest and active participation. This component serves as a mutual supervision mechanism during the preparation phase. It evaluates individual contributions based on task completion, work quality, collaboration, and timeliness. This approach prevents free-riding, ensures accountability, and fosters a collaborative and productive team environment.

The pre-class peer evaluation provides teachers with valuable insights into each student’s level of engagement and contribution within their group. By analyzing the evaluation data, teachers can identify students who may need additional support or motivation, as well as recognize those who excel in leadership and collaboration. This feedback can inform targeted interventions, such as personalized guidance or adjusted group dynamics, to enhance the overall learning experience.

### Inter-group peer evaluation

**Figure 2:**
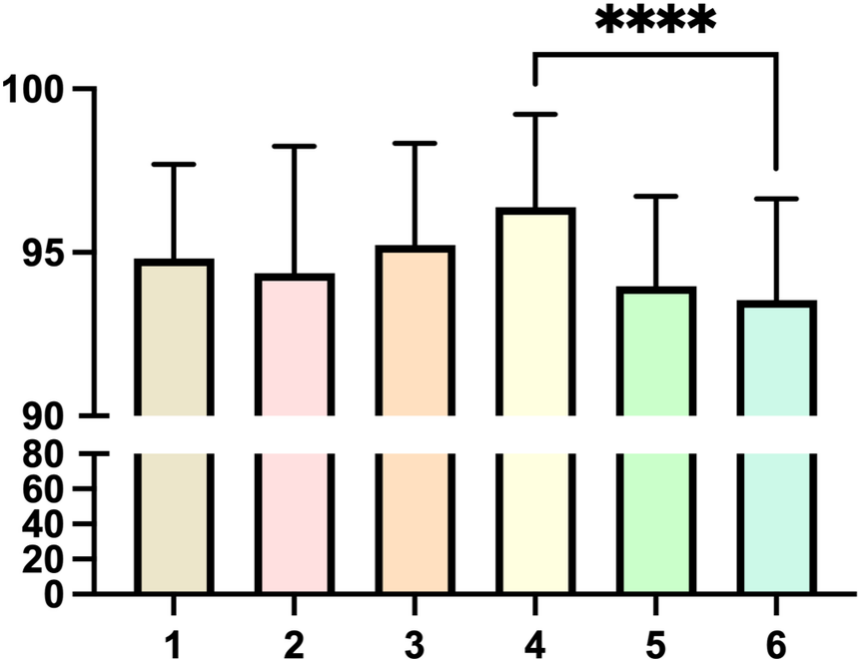
Peer evaluation scores between groups. The figure shows the results of peer evaluation between groups in one class, and there is a significant difference between the highest-scoring Group 4 and the lowest-scoring Group 6, **** *p* < 0.0001.

During the second-class session, each group assigned scores to other groups based on their preparation, presentation format, and overall effectiveness. The evaluation revealed significant differences in scores between the top-performing and the lowest-performing groups (Figure 2), demonstrating a considerable variation in performance among different groups. Notably, faculty assessments demonstrated consistent scoring trends with student evaluations, validating the reliability of the peer-review process. This disparity can be attributed to several key factors: ① case selection: the choice of cases played a critical role in the performance differences. Top-performing groups often selected cases that were highly relevant, engaging, and aligned with the learning objectives, which allowed them to demonstrate a deeper understanding and application of the concepts. In contrast, some lower-performing groups chose cases that were either too complex or not sufficiently challenging, which limited their ability to showcase their skills effectively. ② Group member abilities: the composition and abilities of group members also significantly influenced the outcomes. Groups with members who possessed strong research, organizational, and presentation skills tended to perform better. For example, some groups excelled in live scenario simulations due to members’ strong communication and improvisation skills, while others struggled with video recordings due to limited technical expertise. ③ Preparation levels: the varying levels of preparation among groups further contributed to the performance differences. Top-performing groups invested more time in researching their cases, rehearsing their presentations, and refining their delivery, which resulted in a more polished and impactful performance. On the other hand, some lower-performing groups lacked thorough preparation, leading to less coherent and less engaging presentations. ④Presentation formats: the diverse presentation formats adopted by each group, including live scenario simulations, video recordings and playback, poster design and promotion planning, and charity event planning, also played a role in the observed differences. While some formats, such as live simulations, allowed for dynamic interaction and immediate feedback, others, like video recordings, required more technical proficiency and planning, which not all groups were able to achieve equally.

During the presentation showcase, every student act as a judge, evaluating other groups based on content quality, presentation format, delivery, and overall effectiveness. Students are also required to provide written comments, which encourages active listening, critical thinking, and a deeper understanding of both their own group’s work and that of others.

The inter-group peer evaluation offers teachers a comprehensive view of how students perceive and critique their peers’ work. By reviewing the written comments and scores, teachers can identify common strengths and weaknesses in students’ presentations, such as recurring issues with content organization or delivery. This information can be used to refine teaching strategies and guide students in adopting more effective methods for learning, researching, and summarizing.

In addition to the insights gained from peer evaluations, the study could be further enriched by incorporating teacher-curated case lists. By providing a selection of cases that are equally challenging for all students, teachers can ensure that every student engages with material that is appropriately rigorous. This approach not only levels the playing field but also encourages a more uniform development of critical thinking and analytical skills across the classroom. By curating these cases, teachers can facilitate deeper discussions and more meaningful evaluations, thereby enhancing the overall learning experience for students.

### Questionnaire survey

The third evaluation method employed in this study was a post-class questionnaire survey to collect data. The participants were students from the class of 2021 and 2022 in the clinical medicine program. A total of 331 valid questionnaires were collected and included in the analysis. Before conducting the questionnaire analysis, we performed a Cronbach’s reliability analysis, which yielded a Cronbach’s *α* coefficient of 0.768, indicating that the reliability quality of the research data is good, thus justifying the subsequent analysis (21). Table 2 provides a comprehensive summary of the questionnaire questions and their detailed analysis, highlighting the impact of the PBL teaching method on students’ understanding of congenital malformations. The table is organized as follows:

**Table 2:** Summary of questionnaire questions and detailed analysis.

Question (Q)	Detail analysis
Q1 and Q3: Before and after using the PBL teaching method, how much did you know about congenital malformations? Please select the corresponding range.	Chi-square test showed a statistically significant difference in the data before and after the implementation of the PBL teaching method (*p* < 0.01). The questionnaire survey revealed that prior to the implementation of PBL teaching methodology, students’ understanding of congenital malformations remained inadequate. Specifically, 50.76% of students reported having a relatively basic understanding, while 25.38% expressed ignorance. However, following the adoption of PBL, there was a marked improvement in students’ comprehension of congenital malformations. The percentage of students who were unaware decreased from 25.38% to 4.83%, whereas the proportion of those who understood increased from 17.82% to 54.38%, with 17.22% indicating a profound understanding (Figure 3).
Q2 and Q4: Before and after learning with the PBL teaching method, what common congenital malformations were you aware of?	Q2:Before PBL, students primarily listed 4 common conditions: congenital heart disease, cleft lip and palate, spina bifida, and anencephaly. This suggests a basic but narrow understanding. Q4: After PBL, students listed additional conditions such as neural tube defects, polydactyly, hydrocephalus, and tetralogy of Fallot. This expansion in knowledge breadth demonstrates the PBL method’s ability to broaden students’ awareness of congenital malformations.
Q5: What benefits have you gained from the PBL teaching method?	The survey further indicated that over 70% of students believed that PBL significantly enhanced their teamwork skills, broadened their thinking, and equipped them with a wider range of problem-solving strategies and techniques. More than 50% of respondents acknowledged an increase in their learning interest. Additionally, some students reported improvements in their comprehension, communication skills, as well as their abilities to identify, analyze, and solve problems (Figure 4).
Q6: How satisfied are you with the PBL teaching method for congenital malformations education? Please select the corresponding range.	The survey results demonstrate that the PBL teaching method was highly effective and well-received in congenital malformations education, with 84% of students (Very Satisfied + Moderately Satisfied) reporting positive experiences (Figure 5). The high satisfaction rates reflect the strengths of PBL in fostering active learning, critical thinking, and collaborative skills. However, the feedback from students with neutral or low satisfaction highlights potential areas for improvement, such as providing more structured guidance, enhancing case discussion formats, and offering additional support for students who may struggle with self-directed learning. These insights can inform future refinements to the PBL curriculum to ensure its effectiveness for all students.
Q7: Please rate your mastery of congenital malformations content.	The results of Question 7 demonstrate that the majority of students (89.72%) rated their mastery of congenital malformations content as “Excellent” or “Good,” with an average score of 80. This indicates that the PBL teaching method was highly effective in helping students achieve a strong understanding of the subject. However, the responses from students in the “Pass” and “Fail” categories (10.27%) suggest that additional support and targeted interventions may be necessary to address the needs of those who struggle with the PBL approach or more complex aspects of the content (Figure 6). These insights can guide future refinements to the PBL curriculum to ensure that all students achieve a high level of mastery.

**Figure 3:**
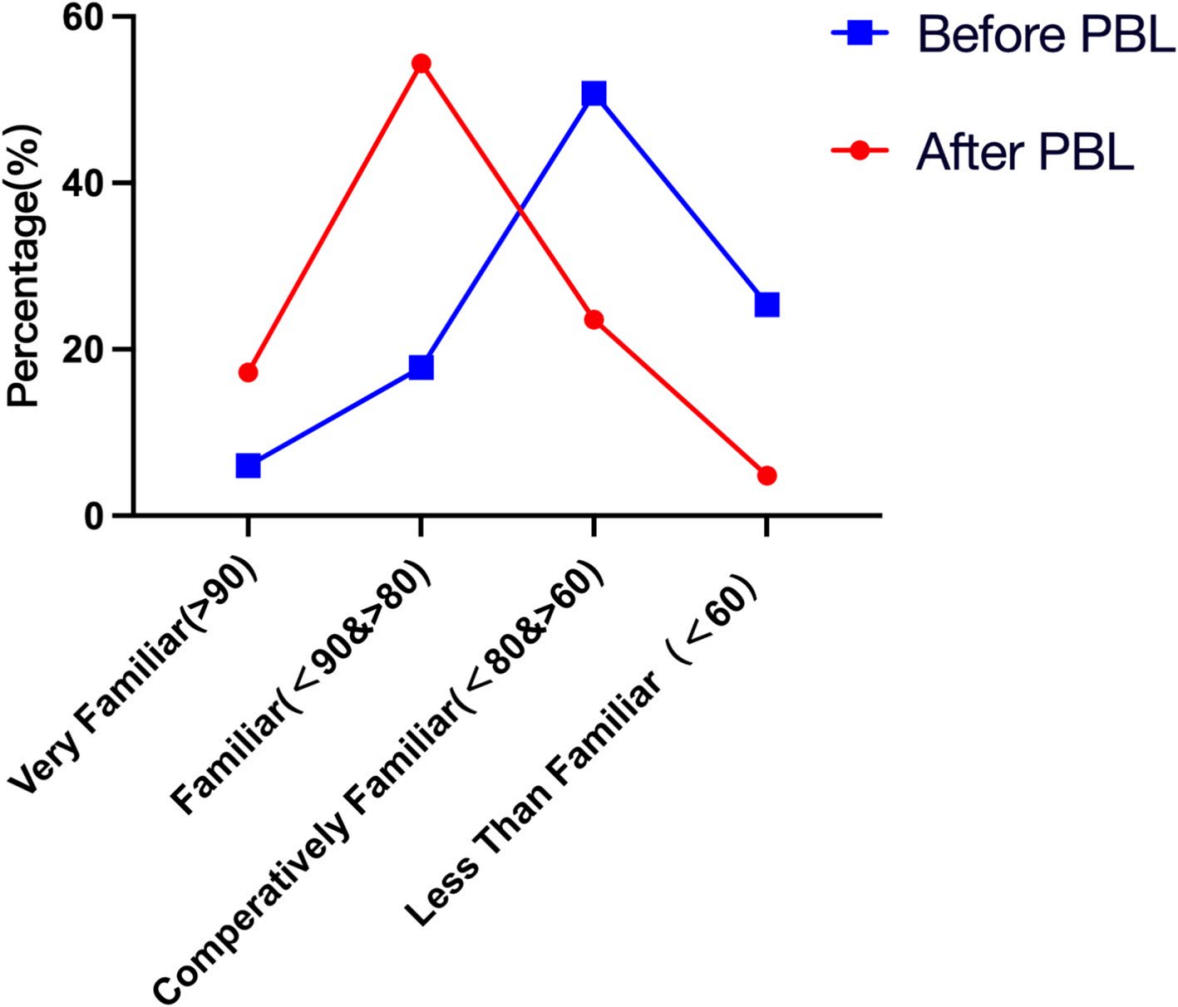
Students’ understanding of congenital malformations before and after using the PBL teaching method. The blue line in the figure represents students’ understanding of congenital malformations before the PBL teaching method, while the red line represents their understanding after the PBL teaching method.

**Figure 4:**
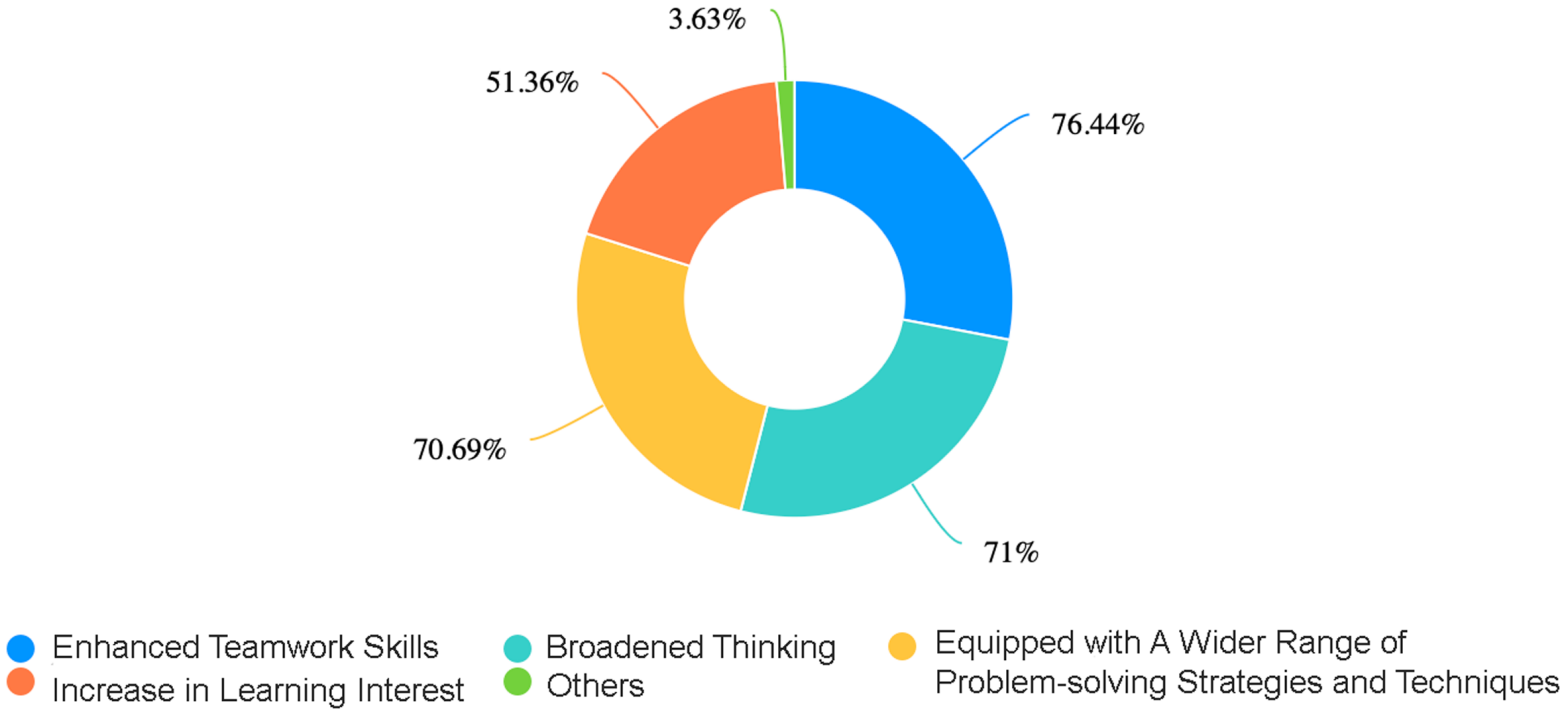
The ring chart presents a distribution of the percentages of the various positive impacts for students after adopting the PBL teaching method. These data outline the percentages of “Enhanced Teamwork Skills,” “Broadened Thinking,” “Equipped with A Wider Range of Problem-solving Strategies and Techniques,” and “Increase in Learning Interest”.

**Figure 5:**
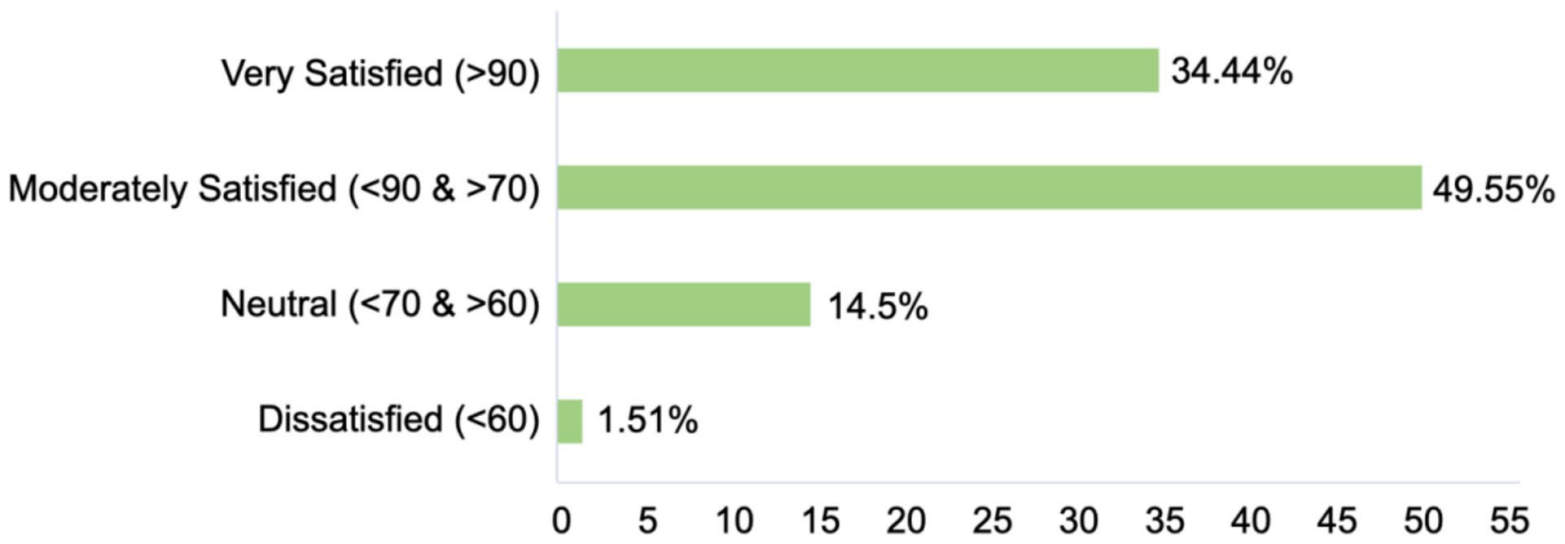
Distribution of student satisfaction with the PBL teaching method in congenital malformations education. The bar chart illustrates the distribution of student satisfaction levels with the PBL teaching method in congenital malformations education. The satisfaction levels are categorized into four groups: very satisfied (>90), moderately satisfied (<90 & > 70), neutral (<70 & > 60), and dissatisfied (<60). The corresponding percentages of students in each category are as follows: very satisfied (>90): (34.44%), moderately satisfied (<90 & > 70): (49.55%), neutral (<70 & > 60): (14.5%), dissatisfied (<60): (1.51%).

**Figure 6:**
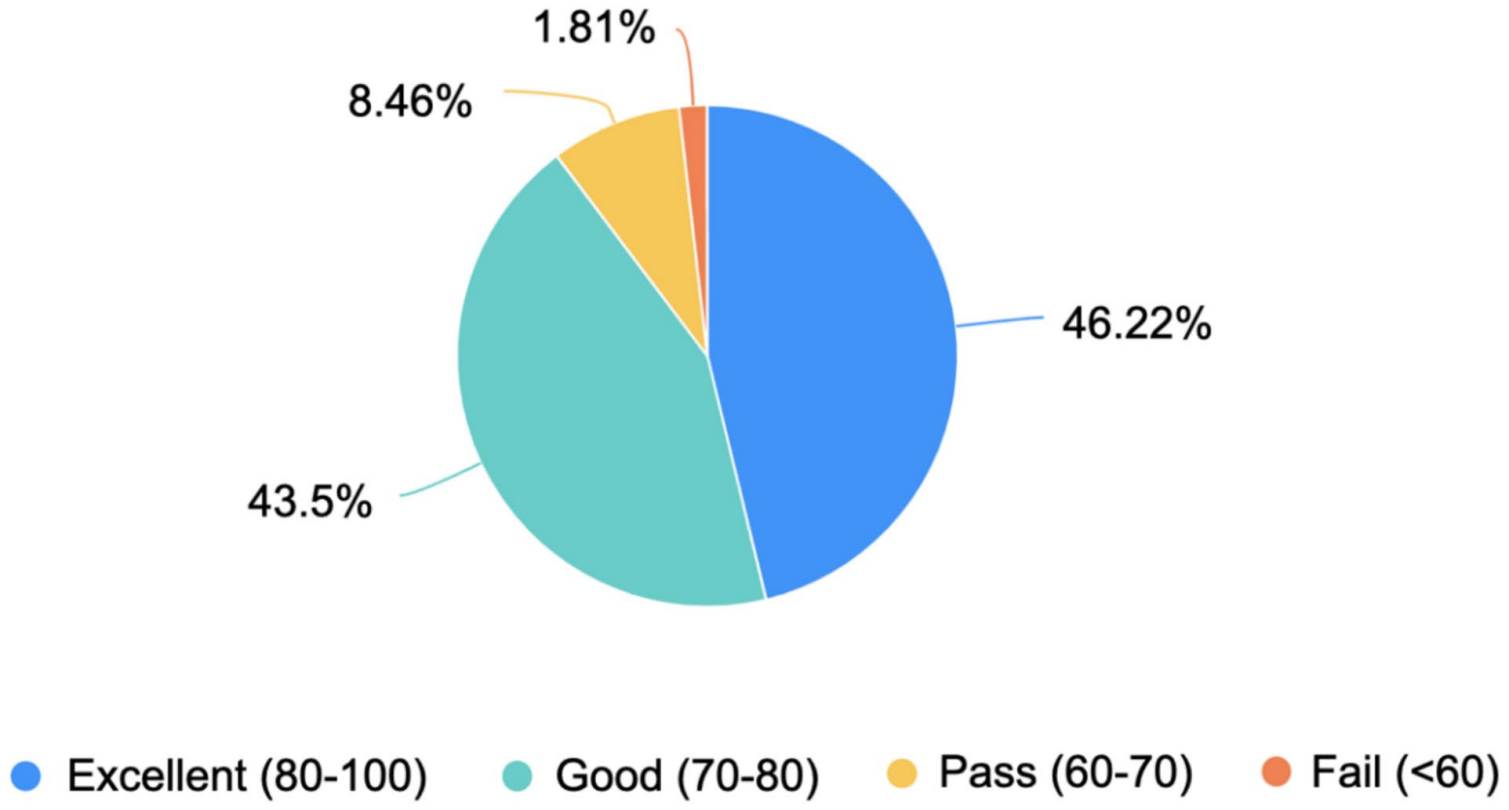
Self-assessed mastery of congenital malformations content among students. The pie chart illustrates the distribution of students’ self-assessed mastery of congenital malformations content, based on a scoring system ranging from 0 to 100. The mastery levels are categorized into four groups: excellent (80–100), good (70–80), pass (60–70), and fail (<60). The corresponding percentages of students in each category are as follows: excellent (80–100): 46.22%, good (70–80): 43.5%, pass (60–70): 8.46%, fail (<60): 1.81%.

These findings underscore the remarkable efficacy of PBL in teaching congenital malformations, a topic characterized by a vast array of types and symptoms. Traditional teaching methods exhibit limitations, as instructors often cover limited content, leaving students in a passive reception role with difficulties in internalizing the knowledge. PBL’s strength lies in its ability to engage students actively through guided case studies, resembling popular narrative-driven puzzle games such as “Murder Mystery Parties” and “Escape Rooms” among young adults, thereby fostering interest and motivation (22–24). Moreover, PBL’s group-based approach fosters teamwork and communication skills as students collaborate to solve problems. By applying their knowledge and skills to real-world scenarios, PBL nurtures students’ divergent thinking and innovative abilities throughout the problem-solving process (Figures 3–6).

## Discussion

Currently, the fact that China’s population growth rate is slowing down objectively exists, yet the number of newborns in 2023 still exceeded 9 million. Understanding congenital malformations, mastering preventive measures against them, and reducing birth defects remain of paramount importance for comprehensively enhancing population quality (3). Therefore, it is still crucial to effectively impart knowledge and skills related to congenital malformations. As a vital component of histology and embryology courses, congenital malformations encompass their causes, prevention, and intersect with various disciplines such as biology, anatomy, genetics, and medicine (25). Traditional teaching methods and limited class hours may fall short in explaining these complex and diverse concepts adequately. However, with the PBL approach, students can integrate and apply knowledge from different disciplines, fostering a more comprehensive understanding and analysis of issues. Additionally, through diverse presentation methods, students can better internalize and assimilate corresponding knowledge. This interdisciplinary learning mode nurtures students’ comprehensive abilities and interdisciplinary thinking.

“Give a man a fish and you feed him for a day; teach a man to fish and you feed him for a lifetime.” PBL transforms the classroom into a proactive arena for exploration and deep diving, where students take the lead while teachers guide their exploration directions. This differs significantly from traditional teaching, which focuses more on imparting fixed and limited knowledge (14). PBL goes beyond that, imparting learning methodologies that encourage students to open their minds, think divergently, and actively seek solutions (26, 27). PBL introduces scenarios through problem formulations, stimulating students’ initiative in learning. With well-crafted cases, students enthusiastically participate, engaging in heated discussions, identifying and posing problems, consulting materials, and resolving issues (14). Their thirst for knowledge is greatly enhanced, and the knowledge acquired through dedicated study is more comprehensive and firmly grasped.

Nevertheless, analyzing student questionnaires and course implementation reveals areas for attention or improvement in applying PBL to congenital malformation teaching. Firstly, PBL poses higher demands on teachers. They must undertake extensive pre-class preparations, whether in preparing cases or guiding discussions, to ensure cases are engaging and motivate students. During classes, teachers must observe students’ performance and promptly offer tailored help and guidance based on their reactions (28). Secondly, PBL presents new challenges for students, requiring them to consult literature, gather materials from diverse sources, discuss in groups, summarize findings, and determine presentation formats and outcomes. This exceeds traditional teaching requirements and necessitates significant after-school efforts. Thirdly, how to ensure that students participate in group discussions and task allocation in a genuine and effective manner, so that every student can actively participate in more in-depth discussions and solve problems more effectively, rather than leaving the task of reporting and presentation to individual group members, requires more effective solutions (29).

### Study limitations and future directions

While this study provides valuable preliminary evidence regarding the effectiveness of PBL in congenital malformations education, several important limitations must be acknowledged along with their corresponding future research directions:

First, the absence of a control group taught through traditional lecture-based methods limits our ability to definitively attribute the observed outcomes solely to PBL pedagogy (30). Future studies should incorporate a randomized controlled design with parallel cohorts receiving conventional instruction to enable direct comparison of relative effectiveness, particularly in measuring knowledge acquisition rates and long-term retention. Such comparative data would substantially strengthen the evidence base for curricular decision-making.

Second, the relatively short duration of the PBL intervention (4 h over 2 sessions) may not have fully captured the potential benefits of this instructional approach. While we observed significant improvements in student engagement and self-reported knowledge, extending PBL implementation across an entire semester with progressively complex cases would allow for more robust development of clinical reasoning skills and better assessment of knowledge retention over time. This extended timeframe would also facilitate the incorporation of spaced repetition techniques known to enhance long-term memory consolidation (31).

Third, our reliance on subjective self-assessment measures, while providing useful qualitative insights, represents a methodological limitation in objectively quantifying knowledge gains. Moving forward, the development and validation of standardized pre- and post-tests specifically targeting congenital malformations knowledge would provide more rigorous quantitative assessment of learning outcomes. These instruments should include both factual recall items and clinical application scenarios to comprehensively evaluate different cognitive domains. Additionally, incorporating objective structured clinical examinations would allow assessment of practical skill development (32, 33).

Moreover, there is limited consideration of confounding factors such as students’ prior knowledge, learning styles, and instructor influence (34–36). It is crucial to investigate whether the observed improvements in student performance could be attributed to the effectiveness of the instructors rather than the PBL approach itself. Addressing these variables in future research will provide a clearer understanding of the factors contributing to student success.

Finally, the study does not discuss whether knowledge retention is sustained beyond the short-term evaluation (31). A follow-up discussion on the long-term application of knowledge in clinical settings would enhance the study (37). Investigating how well students retain and apply their knowledge over time will provide valuable insights into the lasting impact of PBL on their clinical competencies.

The single-institution nature of this study may also limit the generalizability of our findings to other educational contexts with different student demographics or curricular structures. To enhance external validity, future research should employ a multi-center design involving medical schools from diverse geographical regions with varying educational resources (38). This expansion would not only improve the robustness of the findings but also provide valuable insights into how institutional factors may influence PBL implementation outcomes. Such comparative institutional data could inform tailored adaptations of PBL approaches to different educational settings.

The proposed methodological improvements, including controlled comparisons, extended intervention duration, objective assessment tools, and multi-institutional collaboration, will collectively address current study limitations while significantly advancing our understanding of optimal PBL implementation strategies in medical education. By incorporating these enhancements into future research designs, we can generate more robust evidence to inform curricular reforms in both embryology and congenital malformations education.

## Conclusion

This study demonstrates the effectiveness of Problem-Based Learning (PBL) in teaching congenital malformations, highlighting its ability to actively engage students and foster interdisciplinary understanding. By moving away from traditional teaching methods, PBL encourages students to integrate knowledge from various fields, enhancing their critical thinking and problem-solving skills. However, the study also identifies limitations, such as the lack of a control group and the short duration of the intervention, which necessitate further research for more robust conclusions. Future studies should focus on longer-term implementations and standardized assessments to better evaluate knowledge retention and application. Overall, PBL shows promise in improving medical education, particularly in complex subjects like congenital malformations, and warrants further exploration to optimize its effectiveness.

## Data Availability

The raw data supporting the conclusions of this article will be made available by the authors, without undue reservation.
