# Correction: The effect of PBL teaching method in the teaching of congenital malformation

**DOI:** 10.3389/fmed.2025.1649453

**Published:** 2025-07-29

**Authors:** Hua Li, Zejun Cai, Yue Liu, Yong Chen, Qiaomei Lin, Hui Liu

**Affiliations:** ^1^Department of Histology and Embryology, School of Basic Medical Sciences, Fujian Medical University, Fuzhou, Fujian, China; ^2^Key Laboratory of Stem Cell Engineering and Regenerative Medicine of Fujian Province University, School of Basic Medical Sciences, Fujian Medical University, Fuzhou, Fujian, China

**Keywords:** PBL teaching method, traditional teaching methods, congenital malformation, questionnaire survey, evaluation

In the published article, there was an error in Figure 4 as published. The error pertains specifically to the percentage values displayed in the figure. The corrected Figure 4 and its caption “The ring chart presents a distribution of the percentages of the various positive impacts for students after adopting the PBL teaching method. These data outline the percentages of “Enhanced Teamwork Skills,” “Broadened Thinking,” “Equipped with A Wider Range of Problem-solving Strategies and Techniques,” and “Increase in Learning Interest”.” appear below.

**Figure 4:**
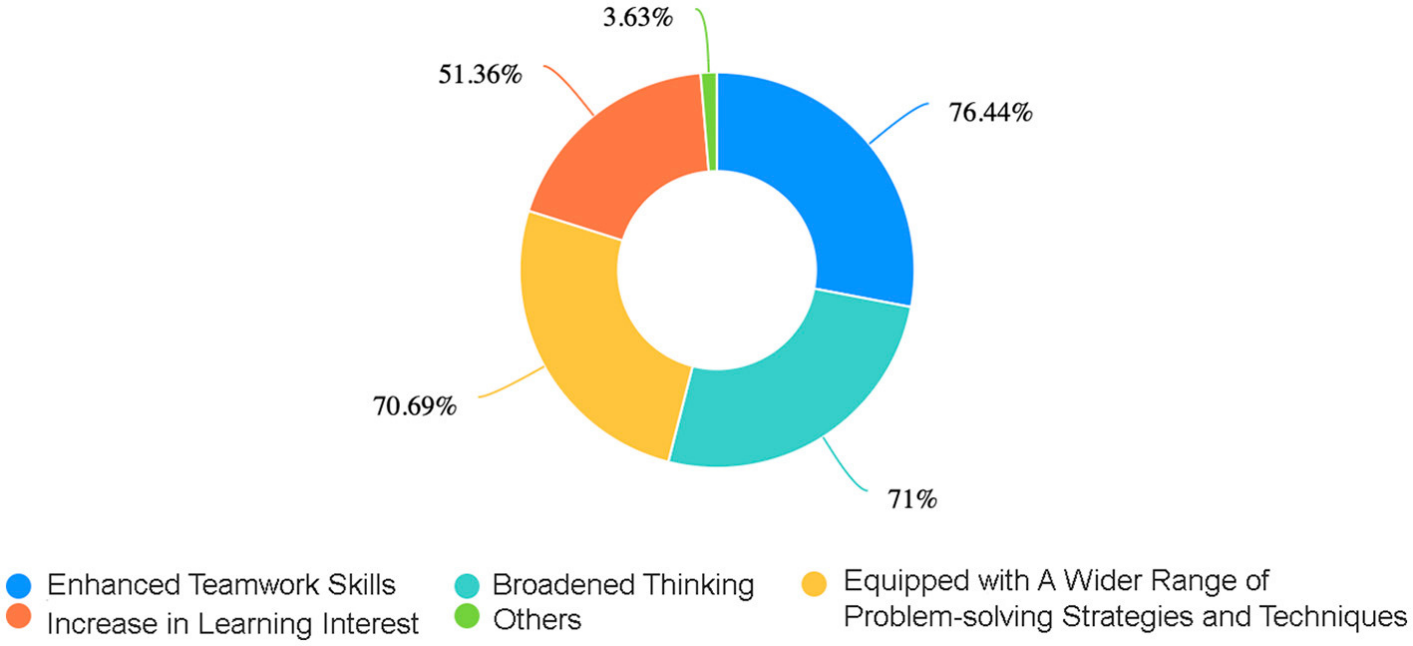
The ring chart presents a distribution of the percentages of the various positive impacts for students after adopting the PBL teaching method. These data outline the percentages of “Enhanced Teamwork Skills,” “Broadened Thinking,” “Equipped with A Wider Range of Problem-solving Strategies and Techniques,” and “Increase in Learning Interest”.

The original article has been updated.

